# Linking a Pharmaceutical Chemistry Workshop to Pharmacy Practice

**DOI:** 10.3797/scipharm.1410-03

**Published:** 2014-12-15

**Authors:** Jordi Morral, Margaret Culshaw, Kim Morral, Barbara Conway, Sylvia Adams, Andrew Adams

**Affiliations:** 1 School of Applied Sciences and Pharmacy, University of Huddersfield, HD1 3DH, United Kingdom; 2 Qualitas Research, Marsden, Huddersfield, HD7 6EL, United Kingdom

**Keywords:** Pharmaceutical Chemistry, Pharmacy Practice, Pharmaceutics, Microbiology, Basic and Applied Science, Community Pharmacy, Dispensary

## Abstract

This paper describes the design and implementation of a workshop to enhance pharmacy students’ appreciation of the importance of chemistry for pharmacy practice. The workshop was designed to form part of the practical work of two modules taught in the second year of the MPharm degree. In this mandatory workshop, second year pharmacy students were required to spot in the dispensary drugs based on their chemical properties like chirality, their origin and chemical structure. The lecturers involved in the workshop showed examples of the application of chemistry in the day to day work of the dispensary (e.g. calculating the dose for a patient in millimoles or how small modifications from a natural product can change its ability to cross the blood-brain-barrier). Feedback from participating students was collected via two survey instruments to examine the impact of the intervention. The survey results showed a clear shift towards a more positive perception by students of the chemistry taught in the MPharm curriculum.

## Introduction

The General Pharmaceutical Council (GPhC) is the independent regulator for pharmacists, pharmacy technicians and pharmacy premises in Great Britain [1]. The GPhC has set out standards for the initial education and training of pharmacists [2], which detail the criteria against which education and training for student pharmacists and pre-registration trainee pharmacists will be approved. An indicative syllabus has been produced which recognises the importance of applied physical, chemical and biological sciences (including pharmaceutical chemistry) [2]. Such recognition is supported by the words of many educators [2]. The MPharm degree in the United Kingdom (UK), as directed by the GPhC guidelines, is a four-year degree of which the last year is at Master’s level. After the MPharm and completion of 52 weeks of assessed *pre*-*registration* training, students have to pass a national registration examination for admission to the GPhC register to be able to work in the UK as registered pharmacists [2]. It has been stated [3], for example, that “a comprehensive understanding of the chemical basis of drug action equips pharmacy students with the ability to answer rationally the ‘why’ and ‘how’ questions related to drug action and it sets the pharmacist apart as the chemical expert among health care professionals”. However, the United Kingdom (UK) pharmacy curriculum model contains a smaller core of chemistry than that of other European universities [4]. Indeed, the principal investigator has worked in pharmacy schools in Spain (Barcelona) and Belgium (Leuven and Antwerp) and is thus aware of the differences in the curriculum in relation to the teaching of chemistry in pharmacy degrees. Furthermore, from the personal experience of the authors, the importance of these sciences is not well understood by a reasonable proportion of students who may share a misconceived perception of the day-to-day work of pharmacists in a community (or hospital) pharmacy dispensary. From our experience, in the UK, we would suggest that some, for example, perceive the pharmacist’s role as largely that of a person who ‘just dispenses medicines’ and, therefore, fail to see the relevance of chemistry to the practice of pharmacy. Further to this, the relevance of medicinal chemistry to pharmacy practice has been questioned by many pharmacy educators [5]. However, more in line with our educational and professional experiences, many other studies in educational research have demonstrated the importance of chemistry to pharmacy practice [5–7].

From the authors’ own experience, informal conversations with colleagues and formal communications with external examiners, it would seem that other lecturers who teach chemistry to pharmacy students have had similar experiences. Some evidence of this comes from the response by pharmacy educators to a recent article in the *Pharmaceutical Journal* (September 2014) [8, 9]. The role of chemistry, and other sciences, in pharmacy practice has recently become a controversial issue in the UK since the Chief Pharmaceutical Officer for Scotland, Bill Scott, textually mentioned in the *Pharmaceutical Journal* (2014; 293: 234) that pharmacy education should “move away from a focus on science”. In the article he asserted that although “we still have the requirement for chemistry”, we “need to move away from that. It’s not about chemistry, it’s about healthcare” [8]. This article in the *Pharmaceutical Journal* generated numerous adversarial responses commenting on the error of these comments [9–14]. Thus, this paper is timely and will be of current interest to pharmacy educators within the UK.

The authors believe that as greater emphasis is placed on linking clinical knowledge and practice to educational outcomes, the pharmaceutical chemistry knowledge of pharmacy graduates diminishes and many students fail to see the interrelation between this subject and other subjects in the MPharm curriculum. In order to address this knowledge gap, the authors of this paper created “The Chemistry of the Dispensary Workshop”, a workshop designed to form part of the practical work of two modules taught in the second year of the MPharm degree at the University of Huddersfield, UK (Pharmaceutical Chemistry II and Pharmacy Practice II), but also incorporating other subjects taught in the MPharm degree including Microbiology, Pharmaceutics, Pharmacology and Pharmacognosy.

The development and implementation of this workshop, together with initial feedback from the participating students, constitutes the subject of this publication. The main idea underpinning the design of the workshop was that, in our opinion, pharmacists may sometimes be unaware of the way in which they use their knowledge of chemistry and other pure sciences in day-to-day professional practice, until they are called upon to communicate this knowledge to other pharmacy staff, healthcare professionals or patients, or to use their specialist knowledge to solve problems that arise in practice. The rationale behind the design and implementation of the workshop was informed by the following objectives:

*Increase student engagement in their learning*.
  The first objective was in recognition of the importance of engagement for effective learning in pharmacy education [15], and more widely [16].
*Enhance student appreciation of the application and relevance of chemistry to the pharmacy dispensary*.
  The second objective was aimed at increasing student acceptance of the Pharmaceutical Chemistry II module of the UK MPharm degree and to show how the material taught in the module has immediate application. This was also with the intention of challenging the aforementioned common misconception of the day-to-day work of the community pharmacist. This objective was considered essential since Pharmaceutical Chemistry II may be the module in which students struggle the most and, despite the extremely applied content of the module, some students question its practical relevance to the world of community pharmacy.
*Increase student awareness of how the subjects of the MPharm degree inter-relate*.
  The GPhC’s standards for the initial education and training of pharmacists [2] states that the curricula must be integrated and the component parts of education and training must be linked in a coherent way referencing the idea of the spiral curriculum [17]. With this in mind, the third objective of the workshop was to try to show second year pharmacy students how the different subjects taught in the MPharm degree are inter-related. To this end, three moderators from different fields of pharmacy (Pharmacy Practice, Pharmaceutics and Chemistry) and a Pharmacy technician supervised the workshop, and the workshop material related to these three subjects (together with some notions of microbiology, pharmacology, pharmacognosy and pharmacokinetics). The three subjects also constituted the academic core of the workshop’s practical booklet, although the emphasis was on Pharmaceutical Chemistry. Similar efforts have been described elsewhere in the areas of physiology [18], biochemistry [19–22] and pharmacology [23], and also (more in line with our work) medicinal chemistry. With regard to the latter, the work of Faruk Khan *et al*. [3] explains how the origins and advancements of pharmacy, medicinal chemistry and drug discovery are interwoven in nature. Of particular relevance is the work developed by Harrold [19] who developed a case study to highlight the importance of functional group chemistry in the drug selection process, with contributions from pharmacology and pharmaceutics faculty members. This was not, however, designed for second year undergraduate students but for more advanced students. The fact that our workshop integrated different disciplines, which is a requirement of the GPhC [2], led us to consider that there was a clear need for the development of such a workshop as an original piece of educational research.

In this respect, this paper is innovative due to its focus on the practical application of chemistry knowledge to the real world, routine work of a community (retail) pharmacist.

Furthermore, we have not come across examples of similar workshops from informal discussions with pharmacy colleagues, or from a review of the relevant literature, and no studies from the UK documenting a workshop of this kind could be found. As described above, research studies that share some similarities with the current one have, however, been developed in other countries such as North America [5–7]. To the best of the authors’ knowledge, no study has previously examined an intervention that sought to increase student awareness of the importance of chemistry in the day-to-day dispensing routines of a community pharmacy.

The workshop required students to use chemistry knowledge, and apply it to pharmacy practice, when performing day-to-day routine tasks such as spotting medicines in a dispensary (for example, reading a drug name and relating the drug name to its chemical structure and stereochemistry) and reading medicine labels (for example, route of administration may be related to the drug salt or ester used). In other related publications [21–26], students had to apply chemical knowledge to pharmacy practice in a more academic (less practical) environment. Furthermore, in this workshop we examined the impact of the workshop on student learning by administrating a questionnaire before and after the workshop.

## Results and Discussion

### Design

The MPharm degree in the UK, as directed by the GPhC guidelines, is a four-year degree of which the last year is at Master’s level. At the University of Huddersfield, Pharmacy Practice is taught in each year of the degree. Pharmaceutics related subjects are also taught each year. Chemistry related subjects, however, are only taught in the first and second years, with only one course. The lead author of this paper is the module leader of Pharmaceutical Chemistry II (now called “Drug Synthesis, Metabolism and Analysis”) which is taught in the second year, and is also a practicing community pharmacist in the UK.

In the UK, the term “dispensary” refers to the place where medicine and medical supplies are dispensed within a community or outpatient pharmacy setting. The workshop was delivered within an adapted classroom at the University of Huddersfield which mimics the function of a real world pharmacy dispensary. The classroom was modified for the purpose of training pharmacists and pharmacy technicians, to equip them with the necessary knowledge related to dispensing and the giving of pharmaceutical advice. This classroom, which is among the university staff and students referred to as “the dispensary”, is equipped with computers, dispensing programs, real medication (*i.e*. registered in the British National Formulary), stocked shelves, a fridge, a controlled drug cabinet and other paraphernalia, with the objective of simulating as closely as possible a real life pharmacy dispensary in a community or hospital pharmacy setup. However, the medication used for training never leaves the classroom.

The desired learning outcome for the workshop was to demonstrate how chemical and scientific knowledge is continuously applied in professional practice. As noted above, three moderators from different fields of pharmacy (Pharmacy Practice, Pharmaceutics and Chemistry) and a pharmacy technician participated in the workshop. They worked alongside students to solve simple chemical calculations and to develop interactive tasks related to the origin and properties of drugs and some notions of formulation.

### Educational environment

The workshop was delivered as part of the MPharm curriculum in February 2013 to second year pharmacy students in the university dispensary. For the workshop, students were divided into two groups, with c.35-40 students in each (77 in total). The workshop involved two identical sessions, with one immediately following the first. Before the workshop, students were asked to read a practical booklet containing material covering the three subjects – Pharmacy Practice, Pharmaceutics and Chemistry – with a focus on Pharmaceutical Chemistry.

The workshop described was originally developed as a one-off, two hour workshop (and can be completed in 90 minutes). This mandatory workshop was designed to consolidate and apply knowledge already acquired in lectures. A booklet explaining the workshop was made available to the students at least one week in advance which they were asked on at least two occasions during lectures to read prior to the workshop.

### Andragogy

The design of the workshop was underpinned by a desire to create a learner focused environment. For this reason, following an introduction to key concepts by the demonstrator, the workshop was divided into two parts: (1) an interactive component focused on four topics (racemates; prodrugs; drug origins; and drug names); and (2) a more academic component on a fifth topic (drugs and their salts).

### Introduction to Key Concepts

At the start of the workshop, the main underpinning concepts which formed the rationale for the workshop’s design and implementation were introduced. Students were first made aware of the different relevance and application of the academic disciplines in Pharmacy. The moderator described how in pharmacy practice we normally concentrate on the drug in use; we consider the patient, the diagnosis, the choice of drug, and the effects of the drug. It was also explained how pharmacists are also concerned with monitoring drug concentration in the body and with a wide range of medicine management issues. In the Pharmaceutical Chemistry module of the MPharm degree students study drug synthesis and metabolism and they are aware that the chemical properties of drugs affect not only the way in which they work and the effects they produce, but also the pharmacokinetic properties and the ways in which they can be formulated. Thus, at the start of the workshop the students were made aware of how the material learnt in Pharmaceutical Chemistry is related to the material learnt in Pharmaceutics, Pharmacokinetics, and even Microbiology. For example, this included the importance of the antibiotic p*K*a being able to reach a particular organ like the prostate, as mentioned by Charalabopoulos *et al*. [24].

Following this presentation of the key concepts, an explanation was then given of how pharmacists are often unaware of the ways in which they use their knowledge of chemistry and other pure sciences in their day-to-day professional practice, until (as noted earlier) they are specifically called upon to share or use their specialist knowledge.

After the introduction by the demonstrator, the main content of the workshop was completed in two parts. The first part of the workshop entailed students identifying in the dispensary a particular type of pharmaceutical specialty while the second part involved a more academic task, requiring students to provide answers to a series of questions. Throughout most of the workshop, students were expected to work in small groups to perform the required tasks.

### Interactive Learning Component

In the first part of the workshop students completed several interactive tasks in the university dispensary. In these interactive tasks, revising material previously taught in lectures, the students were made aware of the following:

- Prodrugs commonly present in a community or hospital dispensary.
- Enantiomer and racemates in pharmaceutical specialties.
- Origins of drugs, natural and semi-synthetic products present in a normal dispensary.
- The importance of polymers.
- How to find the strength of a drug from different salts.
- The use of traditional measures of dose and concentration (including millimoles, mmol) and a new measure of concentration; part per million (ppm).
- How the name of a drug is often indicative of its chemical structure (including functional groups), the chemical structure of its family and its pharmacological activity.

Students were required to identify (spot) in the dispensary a particular type of pharmaceutical specialty related to the subject in question. In this way, students were required to spot medication on the shelves of the university’s adapted classroom that holds real medicines, in a similar manner to what they will encounter in many UK community or hospital pharmacy dispensaries.

When students were expected to identify drugs in common use, which belong to a particular group, demonstrators analysed their findings and provided a context (normally containing anecdotes or stories of potential interest to a pharmacist). The first part of the workshop covered four topics: racemates; prodrugs; drug origins; and drug names.

#### Racemates

The first topic of the workshop was to identify in the dispensary specialties containing drugs that are racemates and explain their meaning. Students were reminded of enantiopure compounds and then shown a specialty containing them such as levothyroxine [25], escitalopram [26] or esomeprazole [27].

#### Prodrugs

The second topic was related to prodrugs and the students had to work in groups to again identify drugs in common use that are prodrugs. Some examples of drugs identified by the students were ramipril [28], clopidogrel [29], folic acid [30] and trazodone [31]. Other examples of prodrugs were provided to the students along with information on the importance of prodrugs in the hospital pharmacy, explaining, for example, the case of cyclophosphamide as a prodrug and mesna as a neutralizer of one of the most toxic of the cyclophosphamide’s metabolites [32].

#### Drug origins

The third topic was related to the origins of drugs and the task was the same as the previous task in which students had to identify in the dispensary specialties that contained drugs that are of natural origin, semi-synthetic drugs and polymers. As before, several examples were given by the students and some additional information was provided to the students about the subject, particularly on hidden natural compounds in pharmaceutical specialties like many excipients such as starches [33] or undecylenic acid [34].

#### Drug names

For the fourth topic, students were asked to identify in the dispensary specialties that contain a drug in which the name is indicative of the chemical structure (e.g. trimethoprim, phenindione, nitrofurantoin, clotrimazole, etc.).

### Academic Component

The second part of the workshop involved tasks of a more academic nature and concerned the fifth workshop topic: drugs and their salts.

#### Drugs and their salts

After providing some background information about the topic, students were asked to perform three tasks. These entailed:

*Task 1*: The first task required students to write the chemical equations of amlodipine maleate and amlodipine besylate when they contact the highly concentrated solution of hydrochloric acid (HCl) in the stomach. After a few minutes, one of the demonstrators explained what happens in the stomach: how in general, when given oral tablets containing different salts, once in our body most of the salts are generally considered interchangeable due to the pH-partition hypothesis [35-37] with some particular exceptions to this rule, like erythromycin [38], also noted.
*Task 2*: The second task was related to the iron content of several iron salts contained in pharmaceutical specialties. The students had the aid of different reference texts such as the British National Formulary (BNF). In the workshop, we highlighted the importance of taking into consideration whether a drug is a salt, a free acid or base. Without some knowledge of chemistry, this concept may be confusing. One example is how the concentrations of phenytoin capsules and tablets and phenytoin suspension [39] are different and how ketamine oral solution is prepared as a hydrochloride but the concentration is expressed as a free base [40]. As before, additional information about the subject, and the correct answer, was provided by the lecturers. The particular case of amoxicillin and clavulanic acid *versus* amoxicillin alone was mentioned, not as an example of different salts used but as an example of how a combination of drugs may increase the power of a particular drug and thus the amount needed may be smaller than expected.
*Task 3:* The third task was related to the different ways of depicting concentrations, such as the use of mmol per litre and parts per million (ppm). As before, students were asked to calculate, in groups, how many mmol of Na^+^ and K^+^ can be found in British specialties containing both ions. The demonstrator remarked on the importance of doses expressed in mmol in the hospital pharmacy when administering ions like potassium [41]. In a similar way, students were told about the use of ppm to express small quantities and how to calculate them taking as an example a British dental specialty that contains NaF expressed in ppm [42].

## Discussion

The educational environment provided by the university dispensary was adequate for the workshop. In the first instance, a circular layout of tables allowed the students to work in groups and facilitated an interactive experience with other students and with the demonstrators. Furthermore, the room was large enough to accommodate a medium sized group of students (up to 48) and all the students had a good view of the dispensary and the wipe board from any seat.

The decision was made to work in groups to promote competition with the idea of engaging the students with the learning experience. In this way no student could avoid interaction with the demonstrators (a situation that can happen in a traditional lecture or even in a laboratory practical set up). Furthermore, due to the circular setting of the workshop, there was no possibility of disengaged students sitting at the back of the class to avoid being asked questions.

The specific details that lead us to the following conclusions are described in the experimental section.

Student response to and engagement in the workshop was positive. According to self-reports of students, collected via two survey instruments, the learning outcomes from the workshop were high. In line with the workshop aims, almost all students reported a greater awareness following the workshop of how chemistry and other sciences are used in the day-to-day professional practice of a pharmacist. Following the workshop, they also had a greater recognition of the need for pharmacists to know more chemistry than other clinical professionals.

In terms of specific learning outcomes, following the workshop many students reported being more aware of how the chemical structure often forms part of the drug name, the presence of products of natural origin in the dispensary, and thus possibly the importance of studying subjects like pharmacognosy. Most students reported that they had learnt a lot about the origin of drugs. All students felt that the workshop had improved their knowledge of the chemical nature and properties of drugs to some degree. Students were a little more aware after the workshop of the need to be able to do simple mathematical calculations to work as a pharmacist, although we believe that many students were already aware of this before the workshop. Following the workshop, more students appreciated the importance of knowing the presentation of a drug (i.e. free base/acid or salt) in order to calculate the dose of a patient.

The survey results revealed two potential areas of greater difficulty to the second year pharmacy students. First, it could be seen that the subject of different drug salts was not well understood, although following the workshop a few students realised that different salts may affect the physicochemical properties of a drug like solubility [43] but once in the organism they do not make much difference. This is because, on the one hand, it is only the portion that is non-ionized that is able to cross biological membranes [35, 44] and, on the other hand, the strongly acidic pH in the stomach will revert salts of acid drugs into the free acid (unless the tablet or capsule is protected) and transform many salts of alkaline drugs (e.g. maleate) into the corresponding hydrochloride and they will be neutralized in the basic pH of the intestine [44]. While it is possible that the questionnaire item was misunderstood by students, we believe that there may have been a lack of basic knowledge about acid base properties and the reactivity of the different functional groups. This idea is backed up by the fact that in other universities these problems may also be present, to the point that a practical aiming to highlight the importance of functional groups has already been developed [19].

Second, while the majority of students were aware following the workshop that there are other units to represent concentration in the dispensary (*i.e*. part per millions, ppm), a reasonable proportion of students (around 20%) did not grasp the idea of the use of ppm to express very diluted concentrations despite the example given. We believe this to be due to a lack of basic mathematical knowledge, or perhaps fatigue, as this part was undertaken near the end of the practical.

On reflection of the findings from both surveys, we believe that this workshop may have opened up some students’ minds and had some effect on changing their opinions regarding the importance and usefulness of chemistry in the day-to-day professional practice of a pharmacist. It would also seem that after the workshop students were more convinced of how chemistry directs the pharmacological properties of drugs. From our observations during the workshop we would suggest that students who showed an awareness of the relevance of chemistry to pharmacy practice were perhaps less skeptical about the worth of learning chemistry in the MPharm degree.

The comments made by students in response to an open ended question in the online survey provide some evidence of student learning from this workshop to pharmacy practice. As described in the results section, a couple of the students commented on how they had gained an appreciation of the relevance of chemistry to pharmacy practice from their participation in the workshop.

It would be methodologically challenging to measure the impact of the workshop on student performance and skills (e.g. to separate the effects of the workshop from the effect of other variables, such as lectures). However, it is worth noting anecdotal evidence that when material that appeared in both the lectures and the workshop were assessed in the final exams, a larger proportion of students answered these correctly, in comparison to questions that only appeared in the lectures.

There was also additional anecdotal evidence of student learning. For example, comments were made a year later, by third year pharmacy students who had participated in the workshop, noting how the workshop had helped them understand the importance of chemistry to pharmacy practice.

The survey results also highlighted the importance of practicals to consolidate knowledge acquired in lectures. Some of the workshop content, such as how the chemical structure often forms part of the drug name, had previously been covered in lectures but it was apparent during the workshop that many students had forgotten some of the material.

## Experimental

### Assessment

To assess the potential of this workshop as a teaching tool data was collected by:

1. A self-completion questionnaire completed by second-year pharmacy students before and following participation in the workshop.
2. An online post-workshop survey of students that took part in the workshop.

#### Pre-and post-workshop survey

One week before the workshop, during the Pharmaceutical Chemistry II lecture, students were asked to complete an anonymous questionnaire related to the topics contained in the workshop. The completed questionnaire was meant to be returned to the lecturers before the workshop started. However, as many students failed to complete and return the questionnaire, immediately before the workshop the same questionnaire was distributed again to the same students to maximize the response rate. At the end of the workshop all students were asked to complete the same questionnaire again.

**Fig. 1:**
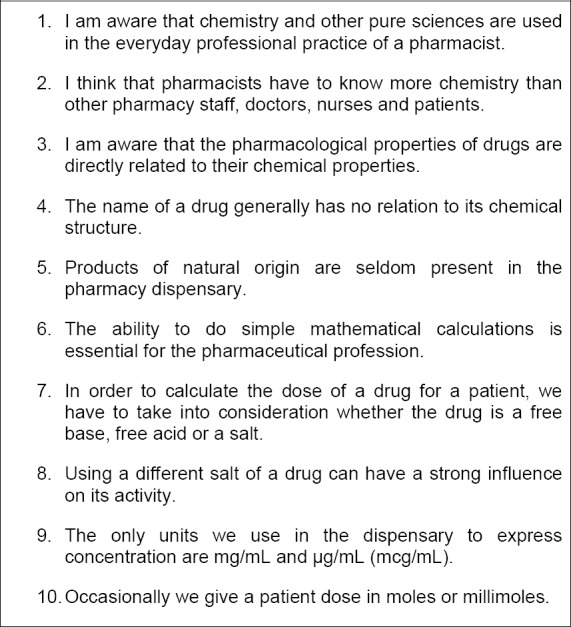
Chemistry of the Dispensary pre-/post-workshop survey instrument.

This short ten-item questionnaire was designed using Likert-style items to assess the impact of the workshop in terms of students’ knowledge and appreciation of how chemistry and other scientific topics are, or can be, applied in the day-to-day work of a pharmacy dispensary, and how the different disciplines they study in the UK’s MPharm degree are interconnected. Students were asked to rate on a five-point scale from strongly agree to strongly disagree how far they agreed with each of ten statements (Figure 1).

There was a high response rate with 66 students (86%) completing the questionnaire before, and 61 (79%) following, the workshop (out of a total of 77 students). The results of the survey are presented in tabular form in Table 1. Within the context of an applied educational project, and with a small sample size, the analysis of survey data was descriptive. Data were analysed using the statistical package SPSS Statistics for Windows, Version 20.0 (2011) to generate frequencies. The analysis was adjusted to take into account the difference in the number of pre- and post-activity respondents.

**Fig. 2:**
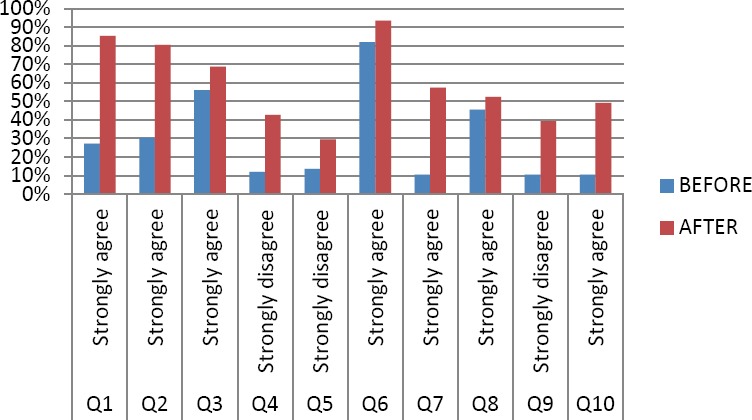
Percentage of students strongly agreeing/strongly disagreeing with statements in the pre-/post-workshop survey.

There was a clear pattern in the survey results for most questionnaire items showing a positive impact of the workshop. As can be seen in Figure 2, there was a distinct shift from students ‘agreeing’ before the workshop to ‘strongly agreeing’ (or ‘disagreeing’ to ‘strongly disagreeing’) with statements after the workshop. From the results of the pre- and post-workshop survey it was evident that the workshop enlightened students in relation to the role of scientific knowledge both in the MPharm degree and the pharmacist’s professional role (*i.e*. the student responses to the survey after the workshop were less scattered and the overall appreciation of the role of chemistry for pharmacists was higher).

The first two questions of the survey instrument assessed students’ knowledge of the role and relevance of chemistry in the professional practice of a pharmacist. More students agreed or strongly agreed with both statements after the workshop. Of interest, there was a marked increase in students *strongly* agreeing with both statements (Q1: 27% strongly agreeing pre-workshop and 85% strongly agreeing post-workshop; Q2: 30% strongly agreeing pre-workshop and 80% post-workshop). There was also an observed positive change for items assessing students’ awareness of how the pharmacological properties of drugs relate to their chemical properties and how the chemical structure of a drug relates to drug names (Q3 and Q4).

Students were next asked if they agreed that ‘the pharmacological properties of drugs are directly related to their chemical properties’ (Q3). While there was no difference in the *total* number of students agreeing/strongly agreeing to this statement before and after the workshop, which was already very high before the workshop (with 98.5% agreeing/strongly agreeing) there was a shift from agreeing to *strongly* agreeing with the statement after the workshop (69% after compared with 56% before the workshop), indicating a perceived positive effect from participation in the workshop.

There was also a shift from ‘agree’ to ‘strongly agree’ (or ‘disagree’ to ‘strongly disagree’) in students’ responses for items assessing students’ knowledge of drugs and their salts (Q7) and their awareness of units of measurement as used in the pharmacy dispensary (Q9). There was a notable increase in the proportion of students who were aware of the use of mmol to express the dose given to a patient (Q10).

There were just two items that yielded little difference in response after the workshop. According to the survey results, there was little change in terms of students’ understanding of the natural origin of drugs (Q5) or students’ awareness of the need for an ability to do mathematical calculations in pharmacy practice (Q7).

#### Post-workshop Online Survey

Following the workshop, all the students who had participated in the workshop were also verbally invited to complete an online survey to give feedback on the experience of participating in the workshop. The online survey was set up on the university intranet by the university’s IT Department and was open for just over six weeks (16 March – 30 April 2011). Students were verbally invited to participate in the survey during the workshop. Verbal reminders to take part were given one week after the workshop during each of the Pharmacy Practice and Pharmaceutical Chemistry II lectures, and again one month later.

The online survey instrument comprised of 10 questions, all but one being closed questions, using a mix of Likert style and dichotomous (yes/no) fixed response questions. Questions asked students to rate the workshop overall, the learning opportunities provided, specific learning outcomes, and whether they would be interested in similar workshops in the future. The online survey was completed by 21 of the 77 students (27% response rate). The results of this survey are shown in Table 2. For the online survey, students were asked to provide feedback, in their own time, for internal evaluation purposes. Despite verbal reminders there was a relatively low response rate (27%) and as such we cannot claim that the responses to the online survey are representative of all participating students.

### Students’ views and opinions of the workshop

Since the response rate to the voluntary online survey was relatively low, a brief summary of results is included here, supplemented by qualitative feedback from students to illustrate the impact of the workshop.

Feedback from students on the workshop was positive (Table 2). Most (86%) of the students rated the workshop as either ‘excellent’ or ‘very good’, and all the students indicated that they would welcome similar integrated workshops. The majority (70%) of students rated the overall learning opportunities provided by the workshop as high. All the students reported that the workshop gave them the opportunity to learn more about the origin of drugs. The majority of students (67%) stated that they felt ‘much more knowledgeable’ about the chemical nature and properties of drugs following the workshop and all felt the workshop had helped them with numerical principles. Importantly, in relation to the workshop objectives, all participating students believed that the workshop helped them to appreciate the overall integration of subject matter across the curriculum.

The online survey included one open-ended question inviting students to comment on any aspect of the workshop with reference to the planning of future workshops. Of the 21 respondents, seven responded to this question. All responses were positive with students describing the workshop as useful, relevant and enjoyable. Comments included:

*I have found the workshop very useful and helpful to us*.

I really enjoyed this – learning can be fun!

Mention was made of the interactive nature of the workshop, the benefit of small group work and also having input from demonstrators from different disciplines. Two students noted, for example:

- I found this workshop more useful than a number of the lectures. The smaller groups with an active involvement and a recognizable relevance to pharmacy practice was especially useful.
- [t] was good having teachers from a range of subjects, definitely need a longer session though. Should have more of these practicals.

The strong link with pharmacy practice was valued and one commented on the advantage of workshops such as this being helpful to students to ‘understand the importance of chemistry in relation to pharmacy’:

- Probably more workshops like the one we had would help students understand the importance of chemistry in relation to pharmacy. It was a great workshop.

In conclusion, the feedback from year two pharmacy students about the Chemistry of the Dispensary Workshop was positive, the workshop was generally well received, and the workshop fulfilled most of our expectations and achieved the goals for which it was designed. In relation to the objectives underpinning the design of the workshop, the workshop had a positive impact on increasing second year pharmacy students’ knowledge of the application of chemistry in the pharmacy dispensary. Importantly, the workshop also appeared to help students to appreciate how the different disciplines studied in the MPharm degree are interconnected.

The main limitation of this study was the implementation of the pre-workshop questionnaire after information had been made available to the participating students. Since the booklet explaining the workshop was made available to the students at least one week in advance of the workshop, and they had been instructed to read this booklet on at least two occasions, we can presume that the students who completed the pre-workshop questionnaire immediately before the workshop had already read the booklet. It seems quite likely that this will have influenced their responses to the pre-workshop questionnaire. Thus, it is possible that the survey results may underestimate the actual impact of the workshop (*i.e*. if the students had been oblivious to the content and objective of the workshop before completing the pre-workshop questionnaire, they may have even been more sceptical about the impact of chemistry in pharmacy practice).

This work was an internal evaluation of a workshop led by a medicinal chemist who is also a registered pharmacist. With regard to future research, it would be interesting if the workshop was replicated and evaluated longitudinally, over two or more years, and with a larger sample of students.

Since its initial implementation in 2011, the Chemistry of the Dispensary Workshop has been repeated annually. There are plans to continue the workshop as part of the MPharm degree at the university in its original format but with some minor modifications to further advance its relevance to pharmacy practice as well as to ensure the content of the workshop is updated in line with developments in community pharmacy practice. One recent addition, for example, is inclusion of the chemistry behind the needle exchange kit for heroin users (the need for purification with citric acid before injecting heroin intravenously).

In terms of future implementation, the full workshop description as used by the University of Huddersfield in the UK is available upon request to the lead author. The workshop can be adapted according to requirement, shortened or lengthened, and by incorporating examples using the national formularies and terminology of different countries.

## Figures and Tables

**Tab. S1:**
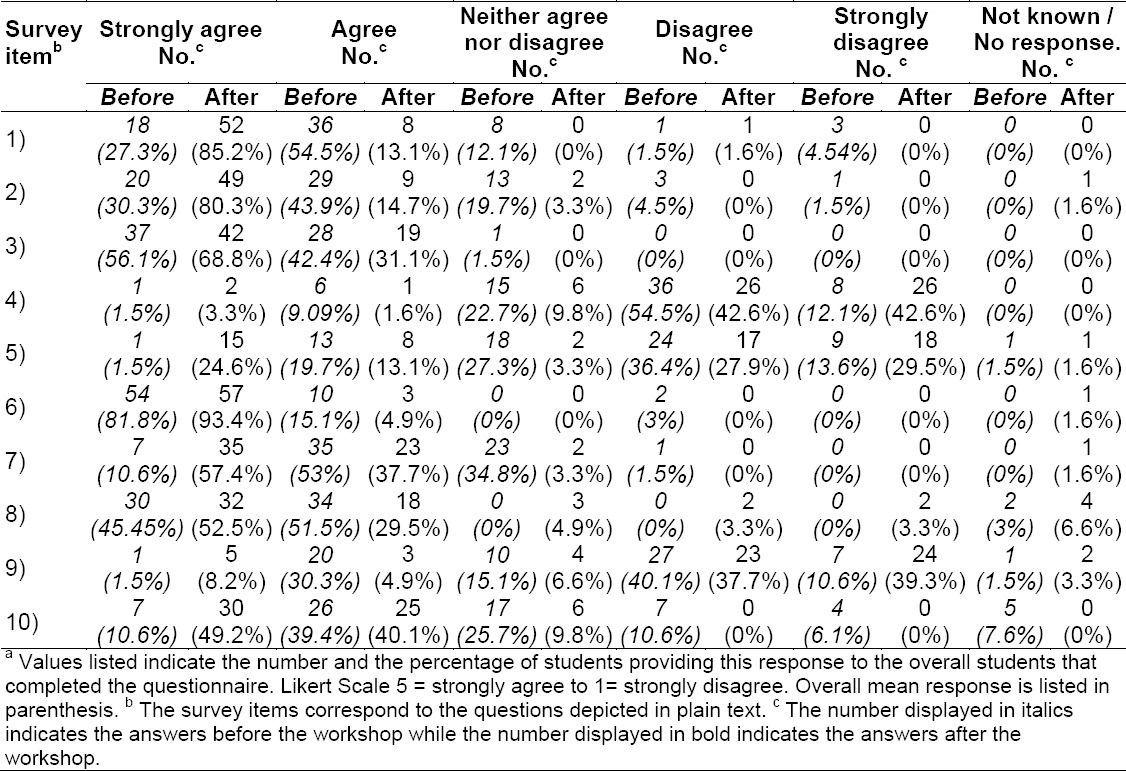
Results of self-completion questionnaire administered to students before and after the workshop (N = 66 before the workshop and N=61 after the workshop)^a^

**Tab. S2:**
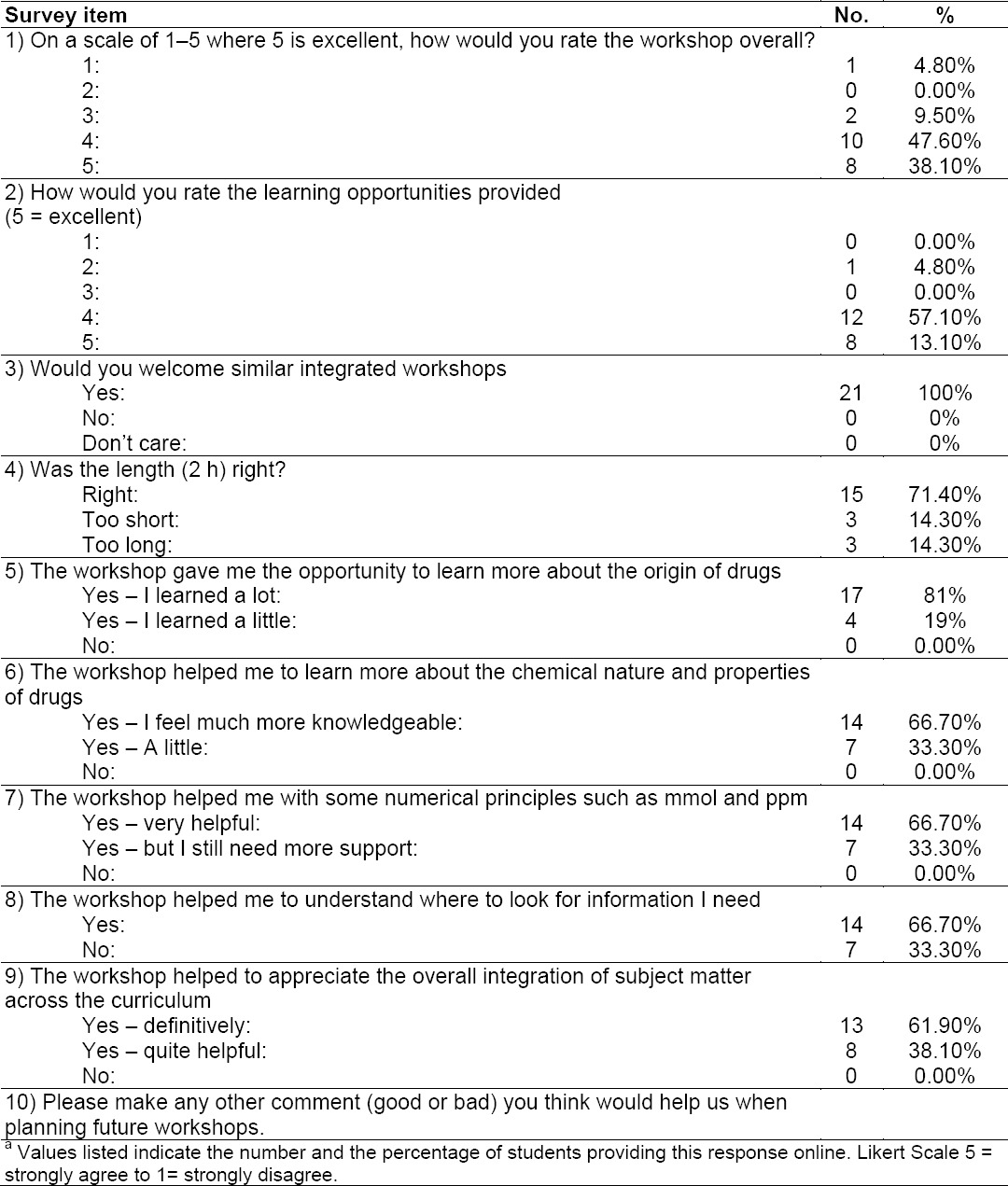
Online post-workshop survey for year-two pharmacy students (N=21)^a^

## Supporting Information

Tables S1 and S2 containing data from the pre-/post-workshop and online student surveys, respectively, are available in the online version (Type: PDF, Seize: < 0.1 MB): http://dx.doi.org/10.3797/scipharm.1410-03.
